# Co-Occurrence of Tetrodotoxin and Saxitoxins and Their Intra-Body Distribution in the Pufferfish *Canthigaster valentini*

**DOI:** 10.3390/toxins12070436

**Published:** 2020-07-03

**Authors:** Hongchen Zhu, Takayuki Sonoyama, Misako Yamada, Wei Gao, Ryohei Tatsuno, Tomohiro Takatani, Osamu Arakawa

**Affiliations:** 1 Graduate School of Fisheries and Environmental Sciences, Nagasaki University. 1-14, Bunkyo-machi, Nagasaki, Nagasaki 852-8521, Japan; zhc957286316@hotmail.com (H.Z.); tfg1akagumi@gmail.com (M.Y.); gaowei0414@hotmail.com (W.G.); taka@nagasaki-u.ac.jp (T.T.); 2 Shimonoseki Marine Science Museum. 6-1, Arcaport, Shimonoseki, Yamaguchi 750-0036, Japan; sonoyama@kaikyokan.com; 3 Department of Food Science and Technology, National Fisheries University, Japan Fisheries Research and Education Agency. 2-7-1, Nagatahonmachi, Shimonoseki, Yamaguchi 759-6595, Japan; tatsuno@fish-u.ac.jp

**Keywords:** tetrodotoxin (TTX), saxitoxin (STX), pufferfish, *Canthigaster valentini*

## Abstract

Pufferfish of the family Tetraodontidae possess tetrodotoxin (TTX) and/or saxitoxins (STXs), but the toxin ratio differs, depending on the genus or species. In the present study, to clarify the distribution profile of TTX and STXs in Tetraodontidae, we investigated the composition and intra-body distribution of the toxins in *Canthigaster valentini*. *C. valentini* specimens (four male and six female) were collected from Amami-Oshima Island, Kagoshima Prefecture, Japan, and the toxins were extracted from the muscle, liver, intestine, gallbladder, gonads, and skin. Analysis of the extracts for TTX by liquid chromatography tandem mass spectrometry and of STXs by high-performance liquid chromatography with post-column fluorescence derivatization revealed TTX, as well as a large amount of STXs, with neoSTX as the main component and dicarbamoylSTX and STX itself as minor components, in the skin and ovary. The toxins were also detected in the other tissues, but in much lower amounts than in the skin and ovary. The TTX/STX ratio varied greatly, depending on the tissue, but TTX was the major toxin component in the whole body, and STXs accounted for 25% and 13% of the total toxin amount in males and females, respectively. Like the marine pufferfish of the genus *Arothron*, *C. valentini* should be considered a pufferfish with considerable amounts of both TTX and STXs present simultaneously.

## 1. Introduction

Pufferfish of the family Tetraodontidae are generally toxic, but the toxin profile differs, depending on the genus or species. Marine pufferfish of the genus *Takifugu* inhabiting the coastal waters of Japan possess the potent neurotoxin tetrodotoxin (TTX) as the major toxic component [1]. The liver and ovary, and, in some species, the skin, are strongly toxic [1,2] and the ingestion of these tissues often causes food poisoning. The muscle and testis, however, are nontoxic or weakly toxic and their consumption is thus permitted in Japan [3,4]. Although the regulatory limits for TTX consumption are not clearly defined in Japan, 10 mouse units (MU)/g tissue (equivalent to 2.2 µg TTX/g or 6.9 nmol TTX/g) is generally used as the criterion for deciding whether or not the pufferfish tissue is edible; the European Food Safety Authority, however, claims that a concentration below 44 µg TTX equivalents/kg shellfish meat, which is much lower than the Japanese criterion, does not result in adverse effects in humans [5]. TTX is an exogenous toxin for pufferfish, which ingest TTX-bearing benthic food organisms such as starfish, marine snails, and flatworms, and accumulate the toxin in specific organs [1]. The intra-body distribution of the toxins may change, depending on the growth and maturity of the individual [6,7,8,9].

However, small-sized freshwater pufferfish of the genera *Pao* and *Leiodon* (both formerly known as *Tetraodon*) living in Southeast Asian countries possess saxitoxins (STXs) instead of TTX as the main toxic component [10,11,12,13]. The skin and ovary are strongly toxic in these pufferfish, whereas the liver toxicity is relatively low. STXs comprise a group of neurotoxins involved in the toxification of bivalves, and the characteristics of the typical component STX are similar to those of TTX, including the molecular weight, toxicity, and intoxication mechanism [14]. Freshwater pufferfish do not live and are not eaten in Japan, but food poisoning, including fatal cases, due to the ingestion of these pufferfish occasionally occurs in Thailand, Bangladesh, and Cambodia [10,11,12,13]. The regulatory limit for STXs set by The Codex Committee on Fish and Fishery Products is 0.8 mg STX·diHCl equivalents/kg tissue [15]. The accumulation mechanism of STXs in freshwater pufferfish is unclear, but it is presumed to be exogenous via the food chain originating from STX-producing cyanobacteria [12].

In general, freshwater pufferfish contain no TTX [10,11,12,13], whereas marine pufferfish not only contain TTX, but also occasionally contain STXs. Even in typical *Takifugu* marine pufferfish, such as *Takifugu pardalis*, *Takifugu flavipterus*, and *Takifugu vermicularis*, trace amounts of STXs may be detected in addition to the main toxin TTX [16,17,18]. Sato et al. [19] investigated the toxin profile of six marine pufferfish species of the genus *Arothron* and *Chelonodon patoca* collected in the Philippines, and reported that they all contain a large amount of STXs in addition to TTX. In particular, in four of the six *Arothron* species, STXs were the dominant toxin rather than TTX. Moreover, Nakashima et al. [20] investigated the toxin profile of *Arothron firmamentum* from Japanese coastal waters, and found that the skin contained only a small amount of TTX, whereas the ovary contained a large amount of toxin, mainly STXs. Therefore, the marine pufferfish of the genus *Arothron* has both TTX and STXs at the same time, with STXs often being the main component, making its toxin profile rather close to that of freshwater pufferfish of the genera *Pao* and *Leiodon*.

**Figure 1:**
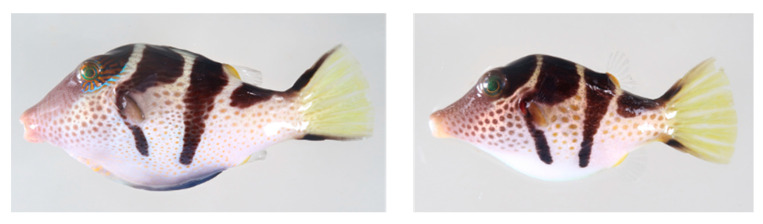
Male (**left**) and female (**right**) specimens of *Canthigaster valentini*. Males can be distinguished from females by the blue-green and orange lines that radiate posteriorly from both eyes, and a blue-gray patch anterior to the anus [25].

*Canthigaster valentini* is a small-sized marine pufferfish that lives on tropical and subtropical coral reefs and has a flashy appearance, with four clear, dark lateral zones on its spotted white body (Figure 1). In an interesting study relating to the appearance, Caley and Schluter [21] analyzed the response of predatory fish to plastic replicas of *C. valentini* (the model fish) and a general fish that looks similar to *C. valentini* (the mimic fish), and suggested that these model-mimic pairs avoid predation by being recognized as ‘toxic fish’ by the predatory fish. Although it is known that *C. valentini* is toxic [22], no information is available regarding the underlying source of its toxicity. Nakatani et al. [23] investigated the toxin profile of a pufferfish of the same genus, *Canthigaster rivulata*, using two individuals of an unknown sex, and detected a trace amount of STXs in addition to the major toxin TTX in the skin. On the other hand, Barrientos et al. [24] detected STXs, mainly comprising STX, with a tiny amount of TTX in *Canthicaster rostrata*, but the distribution of the toxins inside the body is unknown. In the present study, to clarify the distribution profile of TTX and STXs in pufferfish of the family Tetraodontidae, we investigated the composition and intra-body distribution of toxins in *C. valentini*.

## 2. Results

**Figure 2:**
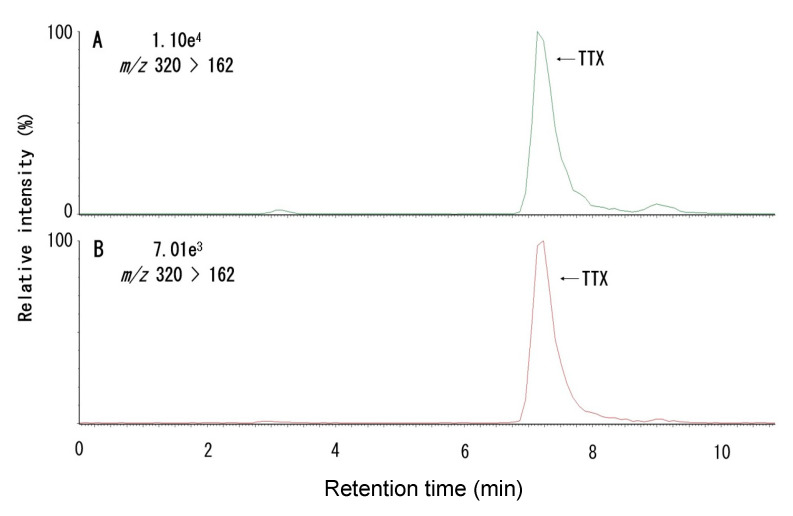
LC-MS/MS chromatograms of the ovary extract from specimen No. 5 (**A**) and of the tetrodotoxin (TTX) standard (**B**).

**Figure 3:**
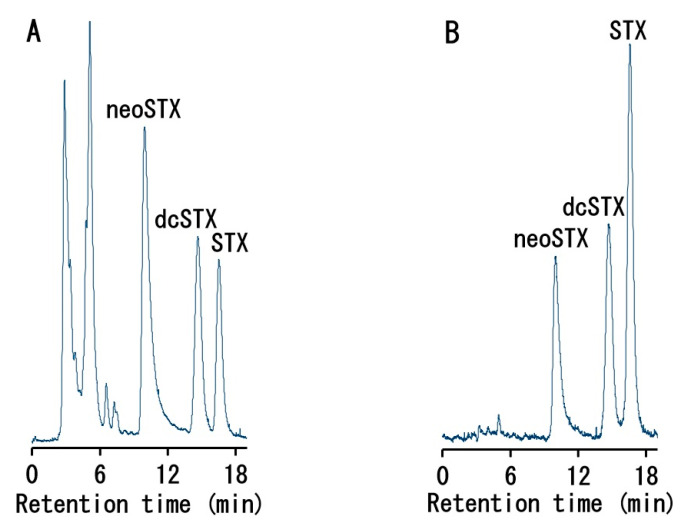
HPLC-FLD chromatograms of the ovary extract from specimen No. 5 (**A**) and of the neoSTX, decarbamoylSTX (dcSTX), and saxitoxin (STX) standards (**B**).

**Figure A1:**
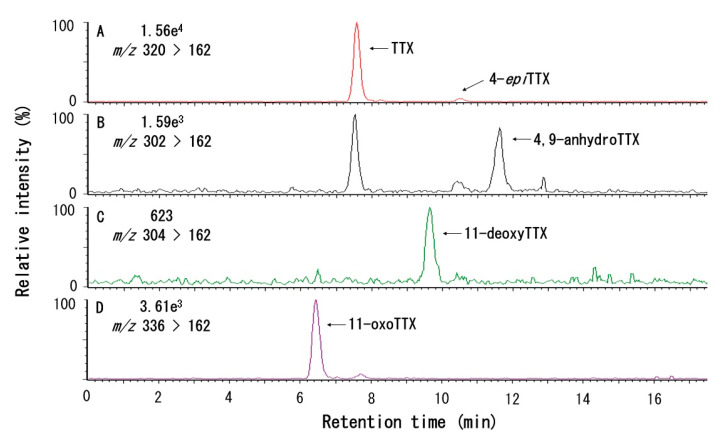
LC-MS/MS chromatograms at *m*/*z* 320 > 162 (**A**), 302 > 162 (**B**), 304 > 162 (**C**), and 336 > 162 (**D**) of the ovary extract from specimen No. 5.

Toxins were extracted from the muscle, liver, intestine, gallbladder, and gonads of four male and six female *C. valentini* specimens (Table 1) collected from Amami-Oshima Island in the Kagoshima Prefecture of Japan. Analysis of the extracts by liquid chromatography tandem mass spectrometry (LC-MS/MS) for TTX [9,26] revealed that samples from all tissues produced a peak with a retention time corresponding to that of the TTX standard in the chromatogram monitored at *m*/*z* 162 as a product ion with *m*/*z* 320 as a precursor ion (described as *m*/*z* 320 > 162 hereafter; a typical chromatogram is shown in Figure 2). Although TTX analogues were not included in the analysis because no standards were available, peaks estimated to be 4-*epi*TTX, 4,9-anhydroTTX, 11-deoxyTTX, and 11-oxoTTX were observed at *m*/*z* 320 > 162, 302 > 162, 304 > 162, and 336 > 162, respectively (typical chromatograms are shown in Figure A1). The peak intensities suggested that 4-*epi*TTX, 4,9-anhydroTTX, and 11-deoxyTTX were present in small amounts, while 11-oxoTTX was present at a higher level. When the same extracts were analyzed by high-performance liquid chromatography with post-column fluorescence derivatization (HPLC-FLD) for STXs [26,27], samples from each tissue, except for the female gallbladder, produced peaks at the retention times corresponding to those of neoSTX, decarbamoylSTX (dcSTX), and STX standards (a typical chromatogram is shown in Figure 3). No known components, such as C toxins and gonyautoxins (GTXs), were detected (data not shown). For the ovaries and skin of specimens No. 5 and 8, the sum of the MU conversion values calculated from the specific toxicity [27,28] of each toxin component (i.e., TTX, neoSTX, dcSTX, and STX) and the toxicity value determined by the mouse bioassay [28] were compared. The two values were in agreement, or the mouse bioassay toxicity value was approximately 10% to 20% higher (Table 2), indicating that more than 80% of *C. valentini*’s toxicity could be explained by TTX, neoSTX, dcSTX, and STX.

**Figure 4:**
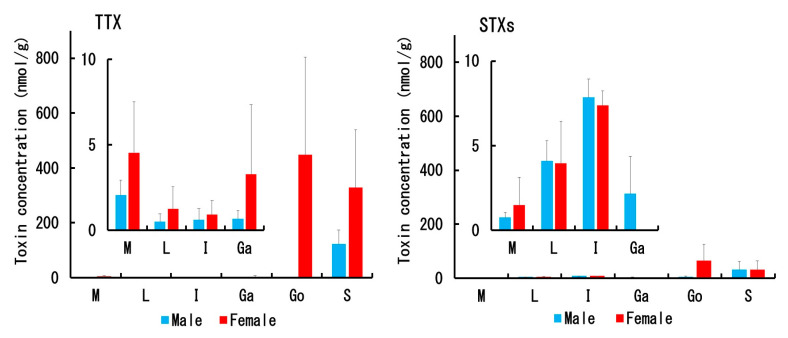
Concentration of TTX (**left**) and STXs (neoSTX + dcSTX + STX; **right**) in each tissue of male and female *C. valentini* specimens. Data are shown as the mean (columns) and SD (error bars). M = muscle, L = liver, I = intestine, Ga = gallbladder, Go = gonads, and S = skin.

The TTX/STX concentration in each tissue of male and female *C. valentini* specimens is shown in Figure 4, and Table A1 and Table A2. The mean TTX concentration of each tissue was generally higher in females than in males. The mean TTX concentration was far higher in the ovary (448 nmol/g) and skin (328 nmol/g) in females and the skin (123 nmol/g) in males, and lower than 5 nmol/g in all of the other tissues, including the liver. On the other hand, the mean STX concentration (sum of neoSTX, dcSTX, and STX) in each tissue was similar for males and females, except for the gallbladder and gonads. Like TTX, the STX concentration was higher in the ovary (65 nmol/g) and skin (30 nmol/g) in females and the skin (30 nmol/g) in males, and less than 5 nmol/g in all of the other tissues except for the intestine (7–8 nmol/g in both sexes). No STXs were detected in the female gallbladder.

**Figure 5:**
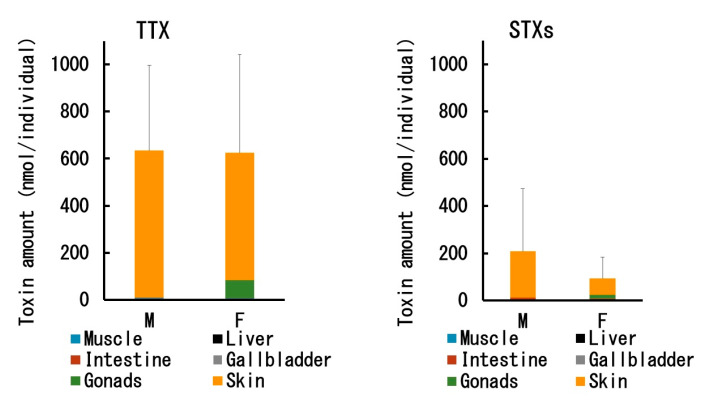
Amount of TTX (**left**) and STXs (neoSTX + dcSTX + STX; **right**) in each tissue of male (M) and female (F) *C. valentini* specimens. Data are shown as the mean (columns) and SD (error bars).

The TTX/STX amount per individual retained in each tissue of male and female *C. valentini* specimens is shown in Figure 5. As described above, the TTX concentration in each tissue was higher in females than in males, but the mean total TTX amount per individual was about the same (~630 nmol/individual) because the individual size of the males was larger than that of the females (Table 1). The skin accounted for most of the total TTX amount in both males and females, although females also had a considerable amount of TTX (76 nmol/individual) in the ovary. On the other hand, the mean total STX amount was higher in males (207 nmol/individual) than in females (93 nmol/individual), and like TTX, most of the toxin was contained in the skin in males, and in the skin and ovary in females.

**Figure 6:**
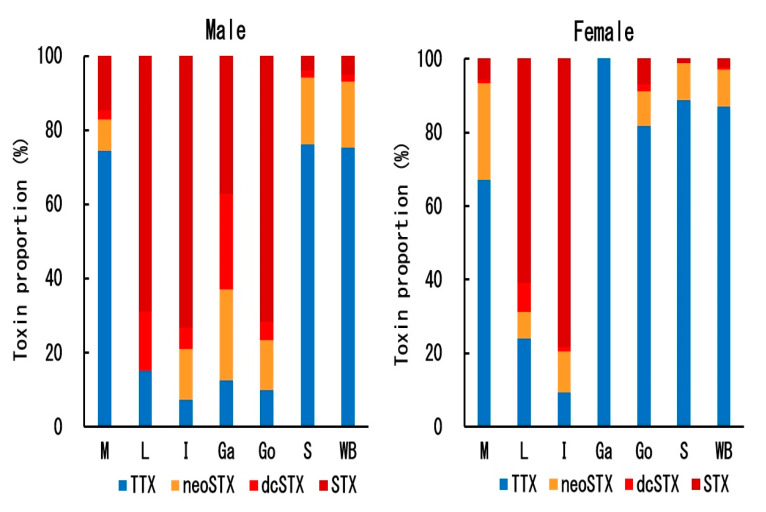
Proportion of each toxin component in each tissue of male (**left**) and female (**right**) *C. valentini* specimens. M = muscle, L = liver, I = intestine, Ga = gallbladder, Go = gonads, S = skin, and WB =whole body.

The proportion of toxin components in each tissue of male and female *C. valentini* specimens is shown in Figure 6. The ratio of TTX and STXs (neoSTX + dcSTX + STX) in both males and females differed greatly, depending on the tissue. In males, TTX accounted for ~75% of the total toxin amount in the muscle and skin, while STXs accounted for ~90% in the other tissues. In females, as in males, TTX was the major component in the muscle and skin, and STXs were the major component in the liver and intestine. In strong contrast to males, TTX was the major component in the gallbladder and gonads in females. The STX composition also differed, depending on the tissue; in the skin and ovary, which accounted for most of the total toxin amount, neoSTX was the main component, and dcSTX and STX comprised the minor components, whereas in the other tissues, except for the female muscle, STX was the main component, and neoSTX and dcSTX were the minor components. In the whole body, the main toxin component of *C. valentini* was TTX in both males and females, but STXs, mainly neoSTX, comprised 25% of the toxin in males and 13% in females.

## 3. Discussion

The present study revealed that *C. valentini* inhabiting Amami-Oshima Island have a large amount of toxin in the skin and ovary. The major toxin component is TTX, but around 20% of the total toxin amount is accounted for by STXs, comprising neoSTX as the main component and dcSTX and STX as the minor components. Various TTX analogues, such as 4-*epi*TTX, 4,9-anhydroTTX, monodeoxyTTXs, dideoxyTTXs, trideoxyTTX, 11-*nor*TTXs, and 11-oxoTTX, have been isolated from several marine pufferfish [29,30,31,32]. As 11-oxoTTX has a stronger toxicity than the other analogues [33,34] and is detected in pufferfish, marine snails, and crabs living in warm seas [33,35,36], its particular contribution to the toxicity of *C. valentini* has been considered to some extent, but as described above, it was excluded from analyses due to the lack of an available standard. On the other hand, an STX component undetectable by HPLC-FLD (carbamoyl-*N*-methyl derivative of STX) was isolated from Bangladeshi freshwater pufferfish [12,37]. Further studies are thus needed to clarify the existence/absence and detailed distribution of such TTX/STX analogues in *C. valentini*.

In *C. valentini,* the skin and ovary were the main toxin-accumulating tissues, and while the other tissues were also toxic, both the toxin concentration and toxin amount were minimal. Hwang et al. [22] examined the toxicity of Taiwanese specimens of *C. valentini* by a mouse bioassay, and reported that only the skin and ovary are toxic (10–200 MU/g). On the other hand, according to Nakatani et al. [23], the closely related species *C. rivulata* contains approximately 40 nmol/g (~60 MU/g) toxin (mainly TTX, along with a small amount of STXs) only in the skin. Therfore, in relation to the toxin distribution in the body, *Canthigaster* pufferfish, although they are a marine species, seem to be closer to freshwater pufferfish of the genera *Pao* and *Leiodon*, with a strong toxicity in the skin and ovary [10,13], than to marine pufferfish of the genus *Takifugu*, with a strong toxicity in the liver and ovary [1,2].

Marine *Takifugu* pufferfish with toxic skin have TTX secretory glands on the skin [38,39], and release TTX in response to external stimuli [40,41]. Itoi et al. [42] observed juveniles of generally nontoxic fish that ingest *Takifugu* pufferfish larvae and then promptly spit them out, and presumed that the TTX transferred from the mother acts to repel predators based on the findings that TTX primarily localized on the body surface of the larvae and that some general fish are able to sense extremely low levels of TTX with gustatory receptors [43]. Although it is currently being investigated whether *C. valentini* have toxin secretory glands on the skin, the extremely high toxicity of the skin strongly suggests that *C. valentini* also use the toxin as a repellent. Moreover, this finding strongly supports the hypothesis of Caley and Schluter [21] that *C. valentini* and their mimic fish avoid predation by being recognized as ‘toxic fish’ by predatory fish. Whether the predatory fish recognize STXs as well as TTX is an interesting issue for future study.

In *C. valentini*, the TTX/STX ratio varied greatly among the tissues; in the skin and ovary, which are the main toxin-accumulating tissues, most of the toxin was accounted for by TTX, whereas in the viscera, such as the liver, intestine, and testis, most of the toxin was accounted for by STXs. Among the six *Arothron* species from the Philippines, whose muscle, liver, intestine, and skin are all highly toxic, one species had a lower skin STX rate than the other tissues and one species had a higher liver STX rate than the other tissues, but the difference was not as extreme as that in *C. valentini* [19]. In the *A. firmamentum* specimens from Japanese coastal waters, in strong contrast to *C. valentini*, STXs accounted for most of the toxin in the ovary, which is the main toxin-accumulating tissue, and only a small amount of TTX was detected in the skin [20]. It is unclear why such a phenomenon occurs, but it may be that TTX/STXs are selectively taken up by the tissues. Nagashima et al. [44] used an in vitro tissue slice incubation method and found that unlike general marine fish, liver slices of *Takifugu rubripes* and marine pufferfish of the genus *Lagocephalus* remarkably take up TTX, but, like general marine fish, hardly take up STXs. The tissue slice incubation method can be applied to other tissues such as the skin and intestine [45], and we plan to use this method to examine the toxin uptake ability or toxin selectivity of each tissue of *C. valentini*.

The STXs harbored by *C. valentini* comprise neoSTX as the main component, and dcSTX and STX as the minor components. STXs harbored by pufferfish usually comprise STX as a main component, and neoSTX as a minor component [10,11,13,16,17,18,19,20,24]. Because STX is the main STX in the intestine, which absorbs the toxin into the body, and in the liver, which is the initial transfer site of the absorbed toxin [7,46,47], it may be that the conversion of STX to neoSTX or selective uptake of neoSTX into the skin and ovary takes place inside the body of *C. valentini*. Further studies are needed to investigate this possibility, together with elucidation of the origin organisms of STXs.

In a recent in vivo TTX/STX administration experiment using nontoxic cultured individuals, Gao et al. [26] found that *T. pardalis*, which possesses TTX in nature, selectively accumulates TTX, and *P. suvattii*, which possesses STXs in nature, selectively accumulates STXs. In other words, the ratio of TTX/STX harbored by pufferfish is presumed to more strongly depend on the inherent TTX/STX selectivity of pufferfish than the prevalence of TTX/STX in prey organisms, but the molecular mechanisms remain to be elucidated. Yotsu-Yamashita et al. [48] isolated a soluble glycoprotein that binds to STX and TTX from the blood plasma of *T. pardalis*, and named it the pufferfish saxitoxin and tetrodotoxin binding protein (PSTBP). PSTBP homologous proteins are widely distributed in toxic pufferfish of the genera *Takifugu* and *Arothron*, but not found in nontoxic pufferfish species or general fish, and are suggested to be involved in the absorption, transportation, and accumulation of TTX in pufferfish [49,50,51]. On the other hand, an ongoing study indicates that freshwater pufferfish of the genus *Pao* do not have the PSTBP gene (Yamada et al., unpublished data). PSTBP also binds to STX [48], but may not be involved in STX accumulation or selectivity. Like the genus *Arothron*, the genus *Canthigaster* could be considered a pufferfish that can simultaneously possess TTX and STXs, but from the viewpoint of the TTX/STX ratio, *Arothron* and *C. rostrata* seem to be closer to *Pao* and *Leiodon*, while *C. rivulata* and *C. valentini* seem to be closer to *Takifugu* marine pufferfish; that is, the STX accumulation ability could increase in the order of *Takifugu*, *C. rivulata/C. valentini*, *Arothron*/*C. rostrata*, and *Pao*/*Leiodon*. The presence or absence of PSTBP homologous proteins in the genus *Canthigaster* and the molecules involved in STX absorption, transportation, and accumulation instead of PSTBP are unknown, but by analyzing and comparing the molecular phylogeny of such toxin accumulation-related proteins with the TTX/STX distribution profile, it may be possible to estimate the evolutionary process in which the pufferfish acquired TTX/STXs. Studies along this line are ongoing.

## 4. Materials and Methods

### 4.1. Pufferfish Specimens

In April 2019, 10 specimens of *C. valentini* (Table 1) were collected at a coral reef off of Amami-Oshima Island in the Kagoshima Prefecture of Japan. They were transported live to the Shimonoseki Marine Science Museum, and after distinguishing males and females on the basis of the male-specific appearance, i.e., blue-green and orange lines that radiate posteriorly from both eyes, and a blue-gray patch anterior to the anus (Figure 1) [25], the muscle, liver, intestine, gallbladder, gonads, and skin were dissected out. The tissues were placed in individual plastic bags, frozen, and transported to the laboratory of Nagasaki University. They were stored below −30 °C until toxin quantification.

### 4.2. Toxin Quantification

Each tissue of the *C. valentini* specimens was extracted with 0.1% acetic acid [28], passed through an HLC-DISK membrane filter (0.45 µm, Kanto Chemical Co., Inc., Tokyo, Japan), and subjected to LC-MS/MS for TTX [9,26] and HPLC-FLD for STXs [26,27].

In the LC-MS/MS analysis, chromatography was performed using an Alliance 2690 Separations Module (Waters, Milford, MA, USA) with a Mightysil RP-18 GP column (2.0 × 250 mm, particle size 5 µm, Kanto Chemical Co., Inc.) and mobile phase comprising 30 mM heptafluorobutyric acid in 1 mM ammonium acetate buffer (pH 5.0) at a flow rate of 0.2 mL/min. The eluate was introduced into a Quattro microTM API detector (Waters) in which the TTX was ionized by positive-mode electrospray ionization with a desolvation temperature of 350 °C, a source block temperature of 120 °C, and a cone voltage of 50 V, and monitored at *m*/*z* 162 (for quantitative analysis) and 302 (for qualitative analysis) as product ions (collision voltage 38 V) with *m*/*z* 320 as a precursor ion through a MassLynxTM NT operating system (Waters). Crystalline TTX (Nacalai Tesque, Inc., Kyoto, Japan) was used as an external standard. The limit of detection (LOD) and limit of quantification (LOQ) of TTX were 0.0009 nmol/mL (*S*/*N* = 3) and 0.003 nmol/mL (*S*/*N* = 10), respectively.

HPLC-FLD was performed using Prominence Ultra-Fast Liquid Chromatography (Shimadzu, Kyoto, Japan) with an LiChroCART Superspher RP18 (e) column (4.0 × 250 mm, particle size 4 µm, Kanto Chemical Co., Inc.). The toxins were separated using three mobile phases: a) 1 mM tetrabutyl ammonium phosphate adjusted to pH 5.8 with acetic acid for C toxins; b) 2 mM heptanesulfonic acid in 10 mM ammonium phosphate buffer (pH 7.3) for GTXs; and c) 2 mM heptanesulfonic acid in 4% acetonitrile–30 mM ammonium phosphate buffer (pH 7.3) for neoSTX, dcSTX, and STX. The flow rate was 0.8 mL/min and the column temperature was 35 °C. The elution from the column was mixed continuously with 50 mM periodic acid and 0.2 M KOH containing 1 M ammonium formate and 50% formamide and heated at 65 °C. The formation of fluorophores was monitored at 392 nm with 336 nm excitation. The reference materials of C1, C2, GTX1-4, and decarbamoylGTX2,3 were provided by the Japan Fisheries Research and Education Agency, and neoSTX, dcSTX, and STX purified from the toxic crab *Zosimus aeneus* [52] were used as external standards. The LOD and LOQ of STXs were 0.001–0.007 nmol/mL (*S*/*N* = 3) and 0.003–0.02 nmol/mL (*S*/*N* = 10), respectively.

In both LC-MS/MS and HPLC-FLD analyses, the analytical range of the standard was up to 1 nmol/mL, and if the toxin concentration of the sample exceeded that range, the sample was re-analyzed after appropriate dilution.

For the ovaries and skin of specimens No. 5 and 8, the sum of the MU conversion values was calculated using the specific toxicity of each toxin component (TTX, 1.45 MU/nmol; neoSTX, 2.30 MU/nmol; dcSTX, 1.27 MU/nmol; and STX, 2.48 MU/nmol) [27,28], and compared with the toxicity value determined by the mouse bioassay described below.

### 4.3. Mouse Bioassay

The ovary and skin extracts of the specimens No. 5 and 8 were examined for their toxicity by a mouse bioassay [28]. The lethal potency was expressed in MU, where 1 MU was defined as the amount of toxin required to kill a 20-g male mouse of the ddY strain within 30 min after intraperitoneal administration. The experiment was performed according to the guidelines for the care and use of laboratory animals set by the Committee on Animal Experiments of Nagasaki University.

### 4.4. Ethical Approval

The mouse bioassay described above was approved by the Committee on Animal Experiments of Nagasaki University (Permission No. 1804021445) (date: 2 April 2018).

## Figures and Tables

**Table 1:** Characteristics of *C. valentini* specimens.

Sex	Specimen No.	Standard Body Length (mm)	Body Weight (g)	Gonadosomatic Index ^1^
♂	1	78.1	32.1	0.19
	2	75.3	25.1	0.05
	3	63.3	13.5	0.13
	4	69.5	17.4	0.17
	Mean ± SD	71.8 ± 6.8	22.0 ± 8.3	0.13 ± 0.06
♀	5	58.1	11.4	2.28
	6	52.4	6.8	1.46
	7	67.9	17.5	2.03
	8	60.9	13.5	1.90
	9	42.9	4.1	0.35
	10	31.1	1.6	0.51
	Mean ± SD	52.2 ± 13.3	9.1 ± 6.0	1.4 ± 0.8

^1^ Gonadosomatic index (GSI), an indicator of maturity, was calculated from the gonad weight (GW) and body weight (BW) using the following equation: GSI = 100 × GW/BW [9].

**Table 2:** Toxicity of the ovary and skin of specimens No. 5 and No. 8 determined by the mouse bioassay and instrumental analyses.

Method	Toxicity (MU/g)			
	Specimen No. 5		Specimen No. 8	
	Ovary	Skin	Ovary	Skin
Mouse Bioassay	1094	720	1223	840
Instrumental Analyses	1100	702	1031	738

## Appendix A

**Table A1:** TTX concentration in each tissue.

Sex	Specimen No.	TTX Concentration (nmol/g)					
		Muscle	Liver	Intestine	Gallbladder	Gonads	Skin
**♂**	1	1.9	0.6	0.6	0.2	0.3	75
	2	3.4	1.1	1.5	0.8	0.8	180
	3	1.5	0.1	0.2	1.3	0.4	151
	4	1.5	0.1	0.01	0.4	0.2	84
	Mean ± SD	2.0 ± 0.9	0.5 ± 0.5	0.6 ± 0.7	0.7 ± 0.5	0.4 ± 0.3	123 ± 51
♀	5	4.0	3.5	2.0	0.8	648	420
	6	2.4	0.2	0.2	1.8	165	108
	7	3.1	0.3	0.4	1.2	414	239
	8	4.3	0.6	0.7	1.2	455	361
	9	3.0	0.6	0.2	2.9	1.1	157
	10	10.4	2.1	1.9	11.5	1005	684
	Mean ± SD	4.5 ± 3.0	1.2 ± 1.3	0.9 ± 0.8	3.2 ± 4.1	448 ± 355	328 ± 211

**Table A2:** STX (neoSTX + dcSTX + STX) concentration in each tissue.

Sex	Specimen No.	STX Concentration (nmol/g)					
		Muscle	Liver	Intestine	Gallbladder ^1^	Gonads ^2^	Skin
**♂**	1	1.0	5.5	8.8	5.3	1.2	78
	2	0.3	3.6	8.5	1.9	-	11
	3	0.9	4.6	7.7	1.4	9.5	10
	4	0.8	2.6	6.3	0	1.5	21
	Mean ± SD	0.7 ± 0.3	4.1 ± 1.2	7.8 ± 1.1	2.1 ± 2.2	4.7 ± 4.0	30 ± 33
♀	5	1.5	3.1	9.0	0	74	40
	6	0.7	2.2	7.5	0	12	4
	7	0.3	5.0	6.7	0	108	13
	8	4.8	7.0	6.9	0	162	94
	9	0.9	0.4	7.3	0	14	21
	10	0.6	6.0	6.7	0	20	10
	Mean ± SD	1.5 ± 1.7	3.9 ± 2.5	7.4 ± 0.8	0	65 ± 61	30 ± 34

^1^ 0: less than the limit of quantification (LOQ), which was considered to be 0. ^2^ -: not examined because of a scarcity of tissue.
